# I FOLLOW THROUGH: CONSCIENTIOUSNESS IS ASSOCIATED WITH ADHERENCE

**DOI:** 10.1093/geroni/igae098.4211

**Published:** 2024-12-31

**Authors:** Sarada Lakshmanan, Sheryl Flynn, Joel Reyes, Stephanie Rubinstein, Elizabeth Zelinski

**Affiliations:** University of Southern California, Los Angeles, California, United States; Blue Marble Health Inc., Altadena, California, United States; Blue Marble Health Inc., Altadena, California, United States; University of Southern California, Los Angeles, California, United States; University of Southern California, Los Angeles, California, United States

## Abstract

Conscientiousness has been linked to positive health benefits, as conscientious individuals have high self-control and are determined to follow a plan (Roberts et al., 2009). Therefore, we tested a sample of 44 older adults aged 57-95, to see whether conscientious individuals were more likely to adhere to a time-intensive, self-guided, digital, home-based fall prevention program. The fall efficacy program contained 11 lessons with 60 5-10-minute-long classes and an avatar-guided program based on the Otago Exercise Program consisting of lower extremity strengthening and balance exercises. Participants completed up to three lessons and exercised up to three days per week. Conscientiousness was assessed using the Big 5 Personality Test. Lesson adherence reflects the number of lessons completed and exercise adherence was measured from baseline to 3 months and from 3 months to 6 months, creating a 3-month and 6-month adherence statistic. We then used Pearson’s r-correlation coefficients to assess the relationships between personality traits and exercise and lesson adherence. None of the traits were significantly related to lesson adherence. Intellect/imagination and extraversion were significant for 3-month exercise adherence (p<.05) while emotional stability was significant for 6-month exercise adherence (p<.05). Conscientiousness was significant for both 3-month (p<.05) and 6-month exercise adherence (p<.05). Conscientious individuals appear to have a stronger tendency to adhere to exercise programs over both 3 and 6 months, suggesting a potential link between conscientiousness and sustained habits. Further analysis is needed to make more definitive and directional conclusions regarding this relationship.

